# Clinical characteristics and outcomes of immunocompromised patients with severe community-acquired pneumonia: A single-center retrospective cohort study

**DOI:** 10.3389/fpubh.2023.1070581

**Published:** 2023-02-15

**Authors:** Xiaojing Wu, Ting Sun, Ying Cai, Tianshu Zhai, Yijie Liu, Sichao Gu, Yun Zhou, Qingyuan Zhan

**Affiliations:** ^1^Department of Pulmonary and Critical Care Medicine, Center of Respiratory Medicine, National Center for Respiratory Medicine, China-Japan Friendship Hospital, Beijing, China; ^2^Capital Medical University, China-Japan Friendship School of Clinical Medicine, Beijing, China; ^3^Graduate School of Peking Union Medical College, Chinese Academy of Medical Sciences, Beijing, China; ^4^Department of Laboratory Medicine, China-Japan Friendship Hospital, Beijing, China

**Keywords:** clinical characteristics, immunocompromised status, intensive care unit, community-acquired pneumonia, risk factor

## Abstract

**Background:**

Immunocompromised patients with severe community-acquired pneumonia (SCAP) warrant special attention because they comprise a growing proportion of patients and tend to have poor clinical outcomes. The objective of this study was to compare the characteristics and outcomes of immunocompromised and immunocompetent patients with SCAP, and to investigate the risk factors for mortality in these patients.

**Methods:**

We conducted retrospective observational cohort study of patients aged ≥18 years admitted to the intensive care unit (ICU) of an academic tertiary hospital with SCAP between January 2017 and December 2019 and compared the clinical characteristics and outcomes of immunocompromised and immunocompetent patients.

**Results:**

Among the 393 patients, 119 (30.3%) were immunocompromised. Corticosteroid (51.2%) and immunosuppressive drug (23.5%) therapies were the most common causes. Compared to immunocompetent patients, immunocompromised patients had a higher frequency of polymicrobial infection (56.6 vs. 27.5%, *P* < 0.001), early mortality (within 7 days) (26.1 vs. 13.1%, *P* = 0.002), and ICU mortality (49.6 vs. 37.6%, *P* = 0.027). The pathogen distributions differed between immunocompromised and immunocompetent patients. Among immunocompromised patients, *Pneumocystis jirovecii* and cytomegalovirus were the most common pathogens. Immunocompromised status (OR: 2.043, 95% CI: 1.114–3.748, *P* = 0.021) was an independent risk factor for ICU mortality. Independent risk factors for ICU mortality in immunocompromised patients included age ≥ 65 years (odds ratio [OR]: 9.098, 95% confidence interval [CI]: 1.472–56.234, *P* = 0.018), SOFA score [OR: 1.338, 95% CI: 1.048–1.708, *P* = 0.019), lymphocyte count < 0.8 × 10^9^/L (OR: 6.640, 95% CI: 1.463–30.141, *P* = 0.014), D-dimer level (OR: 1.160, 95% CI: 1.013–1.329, *P* = 0.032), FiO_2_ > 0.7 (OR: 10.228, 95% CI: 1.992–52.531, *P* = 0.005), and lactate level (OR: 4.849, 95% CI: 1.701–13.825, *P* = 0.003).

**Conclusions:**

Immunocompromised patients with SCAP have distinct clinical characteristics and risk factors that should be considered in their clinical evaluation and management.

## 1. Introduction

Severe community-acquired pneumonia (SCAP) is a common disease in the intensive care unit (ICU), with high mortality rates ranging from 25 to > 50% (1–3). Immunocompromised patients with SCAP warrant special consideration because this population has been growing progressively over recent decades, and an estimated 18–36% of patients with community-acquired pneumonia (CAP) are immunocompromised (4, 5). Among patients with pneumonia, immunocompromised patients experience more severe complications that progress to severe pneumonia (6, 7) and worse outcomes (5).

The clinical characteristics and outcomes of SCAP in immunocompetent patients are well-documented, but few studies have reported on immunocompromised patients with SCAP in intensive care unit (ICU) settings. Most studies of immunocompromised patients with community-acquired pneumonia (CAP) are small, descriptive, and restricted to specific pathogens (such as influenza or bacterial pneumonia) (6, 8), or specific subtypes of immunocompromised conditions (such as human immunodeficiency virus [HIV] or cancer) (9, 10), which limit the generalizability of the conclusions. Delay in initiation of appropriate empiric antibiotic therapy is a known risk factor for worse clinical outcomes, and the etiology of SCAP in immunocompromised patients exhibits different epidemiology, with a greater probability of co-infections; therefore, identifying the causes of SCAP among immunocompromised patients is important. Moreover, immunocompromised patients have mostly been excluded in the published CAP guidelines (11–13) and the characteristics and outcomes of these patients with SCAP are not yet fully understood.

This study aimed to compare the characteristics and outcomes of immunocompromised and immunocompetent patients with SCAP and investigated the risk factors for mortality in immunocompromised patients.

## 2. Methods

### 2.1. Design and population

This single-center, retrospective cohort study was conducted in patients with SCAP, aged ≥ 18 years who were admitted to the ICU of a tertiary academic hospital from January 2017 to December 2019. Patients were excluded if they (1) were suspected of having hospital-acquired pneumonia (14); (2) permanently resided in a nursing home; or if (3) the initial diagnosis of SCAP was not confirmed during their ICU stay.

This study was approved by the institutional review board of the China-Japan Friendship Hospital (2019-80-K52) and was performed in accordance with the Helsinki Declaration. The requirement for informed consent was waived because this non-interventional study collected data from previous electronic medical records and did not involve personal privacy or commercial interests.

### 2.2. Study definitions

SCAP was defined as meeting either one major criterion or at least three minor criteria of the Infectious Diseases Society of America/American Thoracic Society criteria (11). Immunosuppression was defined based on consensus, determined by meeting one of the following criteria (15): primary immune deficiency disease; active malignancy; receiving cancer chemotherapy; HIV infection with CD4 T-lymphocyte count < 200 cells/μL or percentage < 14%; solid organ transplantation; hematopoietic stem cell transplantation; receiving corticosteroid therapy with a prednisone dose of 20 mg or equivalent daily for ≥14 days or a cumulative dose >700 mg; receiving biologic immune modulators; or receiving disease-modifying anti-rheumatic or other immunosuppressive drugs.

Microbiological tests were performed within 48 h of ICU admission in all patients, using bronchoalveolar lavage fluid (BALF) or endotracheal aspirates. Microbiological tests included bacterial and fungal smear and culture, acid-fast stain, and real-time polymerase chain reaction for cytomegalovirus (CMV), *Pneumocystis jirovecii* (PJ), influenza virus, respiratory syncytial virus, adenovirus, *Legionella pneumophila, Chlamydia pneumoniae*, and *Mycoplasma pneumoniae*. Metagenomic next-generation sequencing (mNGS) was performed, if necessary, at the clinician's discretion (more details see Additional file 1).

Pathogens were identified by clinicians based on microbiological tests, clinical manifestations, and chest radiology findings. Atypical pathogens included *Legionella, Mycoplasma*, and *Chlamydia* (16). Polymicrobial infection was defined as having more than one type of pathogen diagnosed by clinicians within 48 h of ICU admission.

### 2.3. Data collection and outcomes

Clinical data, including demographics, comorbidities, season of onset, vital signs, fraction of inspired oxygen (FiO_2_), respiratory support, arterial blood gas analyses, Acute Physiology and Chronic Health Evaluation (APACHE) II scores, Sequential Organ Failure Assessment (SOFA) scores, laboratory results on ICU admission, pathogens detected within 48 h of ICU admission, and clinical outcomes were extracted from the electronic medical record system. The clinical outcomes included the requirement for invasive mechanical ventilation (IMV), IMV duration, length of ICU stay, mortality within 7 days, and ICU mortality.

### 2.4. Statistical analyses

Continuous variables were expressed as means and standard deviations or medians and interquartile ranges. Categorical variables were presented as frequencies and percentages. Differences between characteristics, testing results, pathogens detected, treatments, and outcomes of immunocompetent and immunocompromised patients were tested for statistical significance using *t*-tests for relatively symmetrically distributed continuous variables, Wilcoxon rank-sum test for asymmetrically distributed continuous variables, and Pearson's chi-square test for categorical variables. Two-tailed *P* < 0.05 were considered statistically significant. All statistical analyses were performed using IBM SPSS software, version 22 (IBM Corp., Armonk, NY, United States).

To assess for potential confounders, clinically important variables such as age, lymphocyte count, and CD4 cell count that demonstrated possible statistical associations in the univariate analysis (*P* < 0.1) were transformed from continuous variables into categorical variables according to clinical reference values and standards.

To identify the risk factors of ICU mortality, variables with *P* < 0.1 in the univariate analyses were entered into a multivariable logistic regression model, using stepwise backward selection based on the likelihood ratio. Variable entry was set at *P* < 0.05, and variable removal at *P* > 0.1.

## 3. Results

### 3.1. Study population

Of the 457 patients with SCAP who were screened, 64 were excluded based on the exclusion criteria, leaving a total of 393 patients in the analysis, including 274 (69.7%) immunocompetent and 119 (30.3%) immunocompromised patients. The three most common immunocompromising conditions were corticosteroid therapy (61/119, 51.2%), immunosuppressive therapy (28/119, 23.5%), and active malignancy (16/119, 13.4%) (Supplementary Table S1).

### 3.2. Clinical characteristics and outcomes

Compared to immunocompetent patients, immunocompromised patients included significantly more females (45.4 vs. 30.3%, *P* = 0.004) and patients with chronic lung disease (31.9 vs. 20.8%, *P* = 0.018) (Table 1). The season of disease onset differed significantly (*P* = 0.009) according to immunocompromised status and was mainly in the spring and autumn (49.6%) among immunocompromised patients and the winter among immunocompetent patients (42.3%). The white blood cell (9.1 vs. 10.2, *P* = 0.030), neutrophil (7.9 vs. 8.9, *P* = 0.005), lymphocyte (0.5 vs. 0.7, *P* = 0.044), platelet (150.0 vs. 175.0, *P* = 0.038), and CD4 T cell (201.5 vs. 286.0, *P* = 0.001) counts; and hemoglobin (104.0 ± 21.5 vs. 115.6 ± 25.0, *P* < 0.001), albumin (29.0 vs. 31.0, *P* < 0.001), and procalcitonin (0.8 vs. 1.2, *P* = 0.020) levels on ICU admission were significantly lower in immunocompromised patients than in immunocompetent patients (Supplementary Table S2). Other baseline laboratory results, SOFA scores, and APACHE II scores were similar between patients in the two groups (Table 1). The mean time from SCAP onset to ICU admission in immunocompetent and immunocompromised patients was 8.0 and 7.0 days, respectively (*P* = 0.617).

**Table 1:** The clinical characteristics and outcomes of patients with severe community-acquired pneumonia.

**Variable**	**Total**	**Immunocompromised**	**Immunocompetent**	***P-*value**
	**(*****n*** = **393)**	**(*****n*** = **119)**	**(*****n*** = **274)**	
Age (median, IQR)	63.0 (49.0–73.0)	62.0 (49.0–68.0)	64.0 (50.0–76.0)	0.359
Female (*n*, %)	137 (34.9)	54 (45.4)	83 (30.3)	0.004
Days from illness onset to ICU (median, IQR)	8.0 (5.0–12.0)	7.0 (5.0–13.5)	8.0 (5.0–12.0)	0.617
SOFA score (median, IQR)	6.0 (4.0–9.0)	6.0 (4.0–9.0)	6.0 (4.0–10.0)	0.490
APACHE II score (median, IQR)	19.0 (14.0–24.0)	20.0 (14.0–24.0)	19.0 (14.0–24.0)	0.926
White blood cell ( × 10^9^/L, median, IQR)	9.8 (6.6–14.1)	9.1 (5.9–11.9)	10.2 (6.8–14.6)	0.030
**Season (** ***n*** **, %)**				0.009
Spring and autumn	162 (41.2)	59 (49.6)	103 (37.6)	
Summer	84 (21.4)	29 (24.4)	55 (20.1)	
Winter	147 (37.4)	31 (26.1)	116 (42.3)	
**Comorbidities (** ***n*** **, %)**				
Cardiovascular disease	217 (55.2)	61 (51.3)	156 (56.9)	0.299
Diabetes mellitus	192 (48.9)	55 (46.2)	137 (50.0)	0.491
Chronic kidney disease	32 (8.14)	30 (25.5)	12 (4.4)	< 0.001
Chronic lung disease	95 (24.2)	38 (31.9)	57 (20.8)	0.018
**Positive detection of pathogens (** ***n*** **, %)**	288 (73.3)	99 (83.2)	189 (69.0)	0.003
**Outcome**				
Length of ICU stay (days, median, IQR)	9.0 (6.0–16.0)	8.0 (5.0–14.5)	9.0 (6.0–16.0)	0.182
Duration of IMV (days, median, IQR)	8.0 (4.0–17.0)	5.0 (3.8–12.2)	8.0 (4.0–17.8)	0.176
Using IMV during ICU (n, %)	218 (55.6)	54 (45.4)	164 (60.1)	0.007
Mortality within 7 days (n, %)	67 (17.0)	31 (26.1)	36 (13.1)	0.002
ICU mortality (*n*, %)	162 (41.2)	59 (49.6)	103 (37.6)	0.027

IQR, interquartile range; SD, standard deviation; APACHE, acute physiology and chronic health evaluation scoring system; SOFA, sequential organ failure assessment; IMV, invasive mechanical ventilation; PaO_2_/FiO_2_, the ratio of arterial oxygen partial pressure to fractional inspired oxygen; FiO_2_, fractional inspired oxygen; ICU, intensive care unit.

The use of IMV during ICU stay was less frequent in immunocompromised patients (54/119, 45.4%) than in immunocompetent patients (164/274, 60.1%) (*P* = 0.003); however, the duration of IMV (5.0 vs. 8.0 days; *P* = 0.176) and ICU stay (8.0 vs. 9.0 days; *P* = 0.182) were similar between the two groups (Table 1). The rates of mortality within 7 days (26.1 vs. 13.1%, *P* = 0.002) and ICU mortality (49.6 vs. 37.6%, *P* = 0.027) were higher in immunocompromised patients (Table 1).

### 3.3. Pathogens in patients with SCAP

Pathogens were identified in 83.2% (99/119) of immunocompromised patients and 69.0% (189/274) of immunocompetent patients (*P* = 0.003) (Table 1). Immunocompromised patients had fewer atypical pathogen infections than immunocompetent patients (4.0 vs. 15.3%, *P* = 0.004) (Table 2). Influenza virus infection was more prevalent in immunocompetent patients than in immunocompromised patients (51.3 vs. 15.1%, *P* < 0.001). However, immunocompromised patients had a higher prevalence of infection caused by viruses other than influenza virus (49.5 vs. 12.2%, *P* < 0.001) and cytomegalovirus (47.5 vs. 3.2%, *P* < 0.001) than immunocompetent patients (Table 2). Fungal infections were more prevalent in immunocompromised patients (70.7 vs. 23.3%, *P* < 0.001) (Table 2). Immunocompromised patients had a higher frequency of polymicrobial infections (56.6 vs. 27.5%, *P* < 0.001), especially mixed viral-fungal infections (62.5 vs. 42.3%, *P* = 0.036) (Table 2).

**Table 2:** Characteristics of pathogens in patients with severe community-acquired pneumonia.

**Pathogens (*n*, %)**	**Total**	**Immunocompromised**	**Immunocompetent**	***P-*value**
	**(*****n*** = **288)**	**(*****n*** = **99)**	**(*****n*** = **189)**	
Bacteria	84 (29.2%)	23 (23.2%)	61 (32.3%)	0.109
Viruses	175 (60.8%)	57 (57.6%)	118 (62.4%)	0.423
Influenza virus	112 (38.9%)	15 (15.1%)	97 (51.3%)	< 0.001
No-influenza virus	72 (25.0%)	49 (49.5%)	23 (12.2%)	< 0.001
Cytomegalovirus	53 (18.4%)	47 (47.5%)	6 (3.2%)	< 0.001
Fungi	114 (39.6%)	70 (70.7%)	44 (23.3%)	< 0.001
Aspergillus	67 (23.3%)	23 (23.2%)	44 (23.3%)	0.993
Pneumocystis jirovecii	55 (19.1%)	55 (55.6%)	0 (0.0%)	< 0.001
Atypical pathogens	33 (11.5%)	4 (4.0%)	29 (15.3%)	0.004
Polymicrobial infections	108 (37.5%)	56 (56.6%)	52 (27.5%)	< 0.001
More than one type of bacteria	20 (18.5%)	9 (16.7)	11 (21.1%)	0.497
Mixed bacteria-viruses infection	13 (4.5%)	3 (5.4%)	10 (19.2%)	0.027
Mixed bacteria-fungi infection	9 (3.1%)	3 (5.4%)	6 (11.8%)	0.233
Mixed viruses-fungi infection	57 (19.8%)	35 (62.5%)	22 (42.3%)	0.036
Mixed bacteria-viruses-fungi infection	8 (2.8%)	6 (10.7%)	2 (3.8%)	0.173

**Figure 1:**
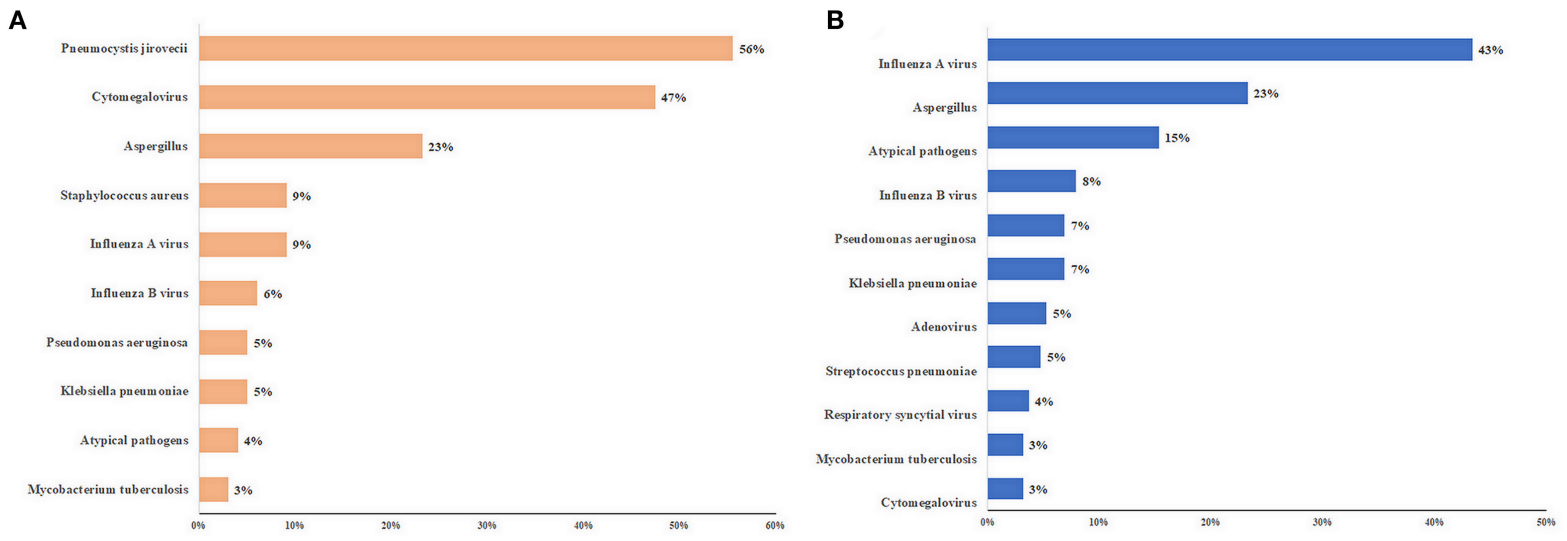
Top 10 pathogens of patients with severe community-acquired pneumonia. **(A)** immunocompromised patients; **(B)** immunocompetent patients. The length of the colored bars and numbers behind them indicate the proportion of each pathogen, calculated using their positive number as the numerator and the number of patients with positive pathogen results as the denominator.

The details of the pathogens identified are shown in Figure 1 and Supplementary Table S3. The five most frequent pathogens identified in immunocompromised patients were PJ (55/99, 56%), CMV (47/99, 47%), *Aspergillus* (23/99, 23%), *Staphylococcus aureus* (9/99, 9%), and influenza A virus (9/99, 9%) (Figure 1). Influenza A virus was the most common pathogen (82/189, 43%) in immunocompetent patients. PJ, nontuberculous mycobacteria (2/99, 2%), and *Nocardia* spp. (2/99, 2%) were detected only in immunocompromised patients. Adenovirus (10/189) and *Streptococcus pneumoniae* (9/189) were detected only in immunocompetent patients (Supplementary Table S3).

### 3.4. Risk factors for ICU mortality

Among immunocompromised patients, the univariate analysis showed that 11 variables were significantly associated with ICU mortality, including age, SOFA score, lymphocyte count, platelet count, D-dimer level, fibrinogen level, PaO_2_/FiO_2_, FiO_2_ group, lactate level, CD4 cell count, and chronic kidney disease (Table 3, Supplementary Table S4). In addition to these variables, the APACHE II score, neutrophil count, and IMV on ICU admission were included in the multivariable logistic regression model (Supplementary Table S4). The final multivariable logistic regression model found that age ≥65 years, SOFA score, lymphocyte count < 0.8 × 10^9^/L, D-dimer level, 0.5 ≤ FiO_2_ < 0.7, FiO_2_ > 0.7, and serum lactate level (were independent risk factors for ICU mortality in immunocompromised patients (Table 4).

**Table 3:** Comparison of clinical characteristics between survival and death immunocompromised patients with severe community-acquired pneumonia.

**Variable**	**Survival**	**Death**	***P-*value**
	**(*****n*** = **60)**	**(*****n*** = **59)**	
Age groups (*n*, %)			0.035
< 45	14 (23.3)	6 (10.2)	
45 ≤ age < 65	28 (46.7)	23 (39.0)	
≥65	18 (30.0)	30 (50.8)	
Male (*n*, %)	25 (41.7)	29 (49.2)	0.412
Season (*n*, %)			0.491
Spring and autumn	33 (55.0)	26 (44.1)	
Summer	13 (21.7)	16 (27.1)	
Winter	14 (23.3)	17 (28.8)	
Days from illness onset to ICU (median, IQR)	8.0 (6.0–13.2)	7.0 (3.5–13.5)	0.171
SOFA score (median, IQR)	4.0 (3.0–8.0)	8.0 (5.0–9.2)	< 0.001
APACHE II score (median, IQR)	17.0 (13.5–23.0)	20.5 (16.0–24.0)	0.059
White blood cell ( × 10^9^/L, median, IQR)	8.8 (5.7–11.4)	9.7 (6.1–12.2)	0.388
Neutrophils ( × 109/L, median, IQR)	7.2 (4.7–9.6)	8.8 (5.5–11.2)	0.076
Lymphocytes < 0.8 × 109/L (*n*, %)	32 (53.3)	47 (81)	< 0.001
Hemoglobin (g/L, mean ± SD)	105.0 ± 20.4	103.1 ± 22.7	0.632
Platelet < 100 × 109/L (*n*, %)	9 (15.0)	25 (42.4)	< 0.001
Albumin (g/L, median, IQR)	29.5 (27.0–32.8)	29.0 (26.0–33.0)	0.823
Creatinine (μmol/L, median, IQR)	70.4 (56.4–98.8)	73.3 (50.5–105.5)	0.191
D dimer (mg/L, median, IQR)	2.1 (1.0–4.3)	4.4 (2.2–11.8)	< 0.001
Fibrinogen (g/L, median, IQR)	6.3 (4.6–7.5)	4.7 (3.6–6.0)	0.006
PaO_2_/FiO_2_ (mmHg, median, IQR)	166.2 (129.7–269.4)	118.4 (90.3–172.6)	0.002
FiO2 groups (*n*, %)			< 0.001
< 0.5	30 (50.0)	10 (16.9)	
0.5 ≤ age < 0.7	15 (25.0)	13 (22.0)	
≥ 0.7	15 (25.0)	36 (61.0)	
Procalcitonin (ng/mL, median, IQR)	0.5 (0.3–2.8)	0.9 (0.6–3.5)	0.034
Lactate (mmol/L, median, IQR)	1.3 (1.0–1.7)	1.7 (1.3–2.3)	0.007
CD4 < 200 cells/mL (*n*, %)	14 (29.8)	33 (63.5)	0.001
IMV on ICU admission (*n*, %)	9 (15.0)	17 (28.8)	0.068
Chronic kidney disease	20 (33.3)	10 (16.9)	0.040
Chronic lung disease	21 (35.0)	17 (28.8)	0.469

IQR, interquartile range; SD, standard deviation; APACHE, acute physiology and chronic health evaluation scoring system; SOFA, sequential organ failure assessment; PaO_2_/FiO_2_, the ratio of arterial oxygen partial pressure to fractional inspired oxygen; FiO_2_, fractional inspired oxygen; IMV, invasive mechanical ventilation; ICU, intensive care unit.

**Table 4:** Risk factors for ICU mortality in immunocompromised patients with severe community-acquired pneumonia.

**Variable**	**OR (95% CI)**	***P*-value**
Age		
< 45	Reference	
45 ≤ age < 65	2.182 (0.425–11.192)	0.350
≥65	9.098 (1.472–56.234)	0.018
SOFA score	1.338 (1.048–1.708)	0.019
Lymphocytes groups ( × 10^9^/L)	
Lymphocyte ≥ 0.8	Reference	
Lymphocytes < 0.8	6.640 (1.463–30.141)	0.014
D dimer (mg/L)	1.160 (1.013–1.329)	0.032
FiO_2_		
< 0.5	Reference	
0.5 ≤ FiO_2_ < 0.7	15.492 (2.252–106.582)	0.005
≥0.7	10.228 (1.992–52.531)	0.005
Lactate (mmol/L)	4.849 (1.701–13.825)	0.003

ICU, intensive care unit; SOFA, sequential organ failure assessment; APACHE, acute physiology and chronic health evaluation scoring system; FiO_2_, fractional inspired oxygen; OR, Odds ratio; CI, confidence interval.

The univariate analysis of ICU mortality in immunocompetent patients revealed 13 variables associated with an increased risk of ICU mortality (Supplementary Table S5). In the multivariable analysis, only 3 factors were independent risk factors for ICU mortality in immunocompetent patients (Supplementary Table S6): days from illness onset to ICU admission (OR: 1.101, 95% CI: 1.039–1.166, *P* = 0.001), platelet count < 100 × 10^9^/L (OR: 2.729, 95% CI: 1.191–6.256, *P* = 0.018), and FiO_2_ > 0.7 (OR: 3.372, 95% CI: 1.433–7.932; *P* = 0.005). Only FiO_2_ > 0.7 was also a risk factor for ICU mortality in immunocompromised patients (OR: 3.372 vs. 10.228 in immunocompetent and immunocompromised patients, respectively).

In the multivariable analysis including all patients, immunocompromised status (OR: 2.043, 95% CI: 1.114–3.748, *P* = 0.021) age ≥65 years, days from illness onset to ICU admission, neutrophil count, platelet count < 100 × 10^9^/L, CD4 T cell count < 200 cells/μL, FiO_2_ > 0.7, and lactate level were independent risk factors for ICU mortality (Table 5). Among them, three risk factors were the same as those for immunocompromised patients: age ≥ 65 years (OR: 4.267 vs. 9.098), FiO_2_ > 0.7 (OR: 4.110 vs. 10.228), and serum lactate level (OR: 1.324 vs. 4.849).

**Table 5:** Risk factors for ICU mortality in all patients with severe community-acquired pneumonia.

**Variable**	**OR (95% CI)**	***P*-value**
Age		
< 45	Reference	
45 ≤ age < 65	1.808 (0.772–4.236)	0.173
≥65	4.267 (1.799–10.117)	0.001
Days from illness onset to ICU	1.076 (1.023–1.132)	0.004
Immunocompromised condition	2.043 (1.114–3.748)	0.021
Neutrophils ( × 10^9^/L)	1.073 (1.024–1.124)	0.003
Platelet ( × 10^9^/L)		
Platelet ≥ 100	Reference	
Platelet < 100	4.187 (2.050–8.551)	< 0.001
CD4 (cells/mL)		
CD4 ≥ 200	Reference	
CD4 T cells < 200	1.921 (1.055–3.497)	0.033
FiO_2_		
< 0.5	Reference	
0.5 ≤ FiO_2_ < 0.7	2.020 (0.958–4.259)	0.065
≥0.7	4.110 (2.057–8.211)	< 0.001
Lactate (mmol/L)	1.324 (1.017–1.724)	0.037

ICU, intensive care unit; FiO2, fractional inspired oxygen; OR, Odds ratio; CI, confidence interval.

## 4. Discussion

This study found that, among patients with SCAP in an ICU, immunocompromised patients had worse clinical outcomes than immunocompetent patients and a large variation in clinical characteristics and pathogen distribution. Additionally, we identified the risk factors of ICU mortality among immunocompromised and immunocompetent patients, which are important for early risk evaluation and may help in developing treatment regimens for these patients. To our knowledge, this study is the first to compare risk factors for mortality among immunocompetent and immunocompromised patients with SCAP.

In this cohort, immunocompromised patients accounted for 30.3% of all patients with SCAP, which is consistent with previous reports (3–5, 17). Although up to one-third of patients with CAP admitted to hospitals worldwide are immunosuppressed, few studies have been conducted among immunocompromised patients, especially those with chronic steroid use and immunosuppressive therapy, and few guidelines are available for managing SCAP in immunocompromised patients. In this study, the two most common immunocompromising conditions were corticosteroid therapy and immunosuppressive therapy. We used a broad definition of immunocompromised status so that our results have broad applicability.

Lower lymphocyte counts, hemoglobin levels, albumin levels, and higher incidence of chronic kidney disease were found in immunocompromised patients, which is consistent with a previous study (18). Compared to immunocompetent patients, immunocompromised patients were more likely to be female, have chronic lung disease, and have lower white blood cell, neutrophil, and platelet counts and procalcitonin levels, which differs from the findings of previous studies (5, 19).

In this study, pathogens were detected in 83.2% of immunocompromised and 69.0% of immunocompetent patients, with more polymicrobial infections in immunocompromised patients than in immunocompetent patients. Our results revealed PJ, CMV, and *Aspergillus* were the three most common pathogens in immunocompromised patients. Influenza A virus was the most common pathogen in immunocompetent patients. Over the past decade, *Streptococcus pneumoniae*, respiratory viruses, *Haemophilus influenzae, Staphylococcus aureus*, and atypical organisms have been frequently identified in patients with SCAP (20). However, few studies have assessed the pathogens among immunocompromised patients with CAP. Bacterial pneumonia accounts for one-third of cases of acute respiratory failure among immunocompromised patients (8, 17). Sousa et al. (5) reported that *Streptococcus pneumoniae* was the most frequent pathogen identified (65.6%), with no differences between immunocompromised and non-immunocompromised patients. In immunocompromised patients with CAP, the most common respiratory pathogens, in addition to the common pathogens of non-immunocompromised patients, include CMV, PJ, *Enterobacteriaceae*, and *Mycobacterium* spp. (21). In this study, a higher proportion of patients had a pathogen identified, and the pathogen distribution differed from those identified in previous studies. These differences may be explained by the choice of microbiological tests and the study population. First, in this study, we employed not only conventional microbiological tests but also mNGS, which has higher sensitivity (22, 23). A previous study found that mNGS may be a useful technique for detecting mixed pathogens in immunocompromised patients with SCAP (24). Second, all included patients completed at least one round of comprehensive microbiological tests within 48 h of ICU admission. Patients were routinely tested for PJ and CMV using BALF. BALF is useful for detecting pathogens in immunocompromised patients with SCAP; the more immunocompromised the patient, the greater the potential benefit (21). Moreover, we used a new, broader definition of immunocompromised status in our study (21), which included a low CD4 count. This could also explain the different pathogen distribution reported in the present study because different immunocompromising conditions may be associated with different pathogen susceptibility (4, 8, 18, 21). Another possible explanation of why our results differed from those of previous studies is that the median time from illness onset to ICU admission was 8 days, and most patients had prior antibiotic exposure before ICU admission, which might have resulted in a lower bacteria detection rate.

Immunocompromising conditions are reportedly associated with mortality in patients with influenza (6, 7), CAP (5, 19), and community-acquired respiratory virus infections (18). Our results are consistent with those of previous studies showing that immunocompromised patients with SCAP had higher early mortality (within 7 days) and ICU mortality. Moreover, immunocompromising conditions and CD4 count < 200 cells/μL were the two independent risk factors for ICU mortality in patients with SCAP, which can add important evidence to previous information.

The multivariable logistic regression analysis of ICU mortality in immunocompromised and immunocompetent patients revealed significantly different risk factors between the two groups; FiO_2_ > 0.7 was the only common risk factor. Clinicians should consider these different risk factors for early diagnosis and management of SCAP. Few studies have examined the risk factors for poor outcomes in immunosuppressed patients with CAP or severe pneumonia, and previous studies have had inconsistent results (6, 10, 25, 26). Some independent risk factors for ICU mortality in immunocompromised patients with SCAP, such as age, SOFA score, FiO_2_ > 0.7, and D-dimer and lactate levels, have been reported previously (26–29). Lymphocyte count < 0.8 × 10^9^/L was also a risk factor for these patients. Chen et al. (6) found that baseline lymphocyte counts of 0.6 × 10^9^/L were independently associated with 30-day mortality in immunocompromised patients with influenza-related pneumonia. Lymphocytes are closely related to the immune function in pneumonia and can be recruited to the respiratory tract and reduced in circulation. Guo et al. (30) reported that lymphocyte count ≤ 0.8 × 10^9^/L is one of the predictors of mortality in patients with viral pneumonia. Moreover, PJ and CMV are the most common pathogens in immunocompromised patients. Previous studies have identified lymphocyte count as a risk factor that can influence the mortality of PJ (31) and viral pneumonia (19).

Our study has several limitations. First, it was a small, single-center study and thus susceptible to selection bias. Half of the immunocompromised patients received corticosteroid therapy, and so the immunocompromised patients may not be representative of all potentially eligible immunocompromised patients. Second, given its retrospective design, pathogen tests and diagnoses were conducted based on the clinician judgment and ICU routine, without standardized criteria. Moreover, most patients had prior antibiotic exposure before ICU admission, which might have resulted in the low detection rate of bacteria. Finally, our results are limited to patients with SCAP that necessitated ICU admission and may not be applicable to all patients with SCAP.

## 5. Conclusion

Compared to immunocompetent patients with SCAP, immunocompromised patients with SCAP have distinct clinical characteristics and pathogen distributions. Immunocompromised status is an independent risk factor for ICU mortality in patients with SCAP. These findings can be used to guide clinicians in evaluating and managing immunocompromised patients with SCAP in the ICU setting.

## Data availability statement

The raw data supporting the conclusions of this article will be made available by the authors, without undue reservation.

## Ethics statement

The studies involving human participants were reviewed and approved by China-Japan Friendship Hospital (2019-80-K52). Written informed consent for participation was not required for this study in accordance with the national legislation and the institutional requirements.

## Author contributions

QZ provided the study concept, design, and contributed to subsequent versions. XW, TS, YC, TZ, YL, SG, and YZ collected the data. YL and TS performed the statistical analyses. TS and XW drafted the manuscript. All authors have read and approved the final manuscript.
