# Supplementary material for: Clinical characteristics and outcomes of immunocompromised patients with severe community-acquired pneumonia: A single-center retrospective cohort study
Source: Front Public Health. 2023 Feb 15;11:1070581. doi: 10.3389/fpubh.2023.1070581 (PMC9975557; doi:10.3389/fpubh.2023.1070581)
Supplement: Supplementary file 1 [file Data_Sheet_1.docx]

**Additional File Content**

**Clinical characteristics and outcomes of immunocompromised patients with severe community-acquired pneumonia: a single-center retrospective cohort study**

**Additional file 1:** Additional information of identification of pathogens

**Supplementary Table S1** Immunocompromising conditions of immunocompromised patients

**Supplementary Table S2** The laboratory testing results of patients with severe community-acquired pneumonia (immunocompromised vs immunocompetent patients)

**Supplementary Table S3** Distribution of pathogens in patients with severe community-acquired pneumonia (immunocompromised vs immunocompetent patients)

**Supplementary Table S4** Univariable analysis of ICU mortality in immunocompromised patients with severe community acquired pneumonia (Variables entered the multivariate logistic regression model with *P* values < 0.1)

**Supplementary Table S5** Univariable analysis of ICU mortality in immunocompetent patients with severe community acquired pneumonia (Variables entered the multivariate logistic regression model with P values < 0.1)

**Supplementary Table S6** Risk factors for ICU mortality in immunocompetent patients with severe community acquired pneumonia

**Additional file** **1** **Additional information of identification of pathogens**

The clinicians will confirm the pathogen etiology based on clinical manifestation, laboratory examination, chest radiology, and microbiological results (including conventional microbiological tests and metagenomic next-generation sequencing) (1, 2). Microorganisms with unclear significance, such as Torque teno virus (TTV) and EBV, were considered “non- pathogenic microbes.” Oral and intestinal microorganisms were identified as colonized or contaminated unless clinical evidence is available, such as aspiration.

The conventional microbiological tests included bacterial or fungal smear and culture, acid-fast stain, and real- time polymerase chain reaction (PCR) to detect cytomegalovirus (CMV), influenza virus, respiratory syncytial virus, Pneumocystis jirovecii, Legionella, Mycoplasma, and Chlamydia. PCR was performed at the clinician’s discretion. Previous studies have reported that the CMTs of BALF samples (smear, culture, and PCR) were consistent with the standard clinical procedure.

Given the lack of standard threshold criteria for interpreting Metagenomic next-generation sequencing (mNGS) results, we used the criteria derived and revised from previous studies(1, 3-6).The mNGS results were considered as positive if one of the following thresholds were met, and a literature evidence of its pulmonary pathogenicity is available: (i) culture/PCR and mNGS identified the same microbe; (ii) at the species level, the relative abundance of bacteria (mycobacteria excluded), fungi (molds excluded), and parasites was greater than 30%, or the coverage rate scored 10-fold greater than that of any other microbes (for fungi, whose coverage rate scored 5-fold higher than that of any other fungus); (iii) positive virus and molds will be considered when the SDSMRN was no less than 3; (iv) for the detection of Mycobacterium spp., Nocardia spp., the pathogen was considered as positive when SDSMRN is greater than 1.

**Table S1** Immunocompromising conditions of immunocompromised patients

|  | |  |
| --- | --- | --- |
| **Patient ID** | **immunocompromising conditions** |  |
| 5 | Active malignancy |  |
| 7 | Immunosuppressive therapy |  |
| 11 | Immunosuppressive therapy |  |
| 21 | Corticosteroid therapy |  |
| 28 | Cancer chemotherapy |  |
| 30 | Immunosuppressive therapy |  |
| 31 | Corticosteroid therapy |  |
| 32 | Immunosuppressive therapy |  |
| 34 | Corticosteroid therapy |  |
| 35 | Immunosuppressive therapy |  |
| 37 | Corticosteroid therapy |  |
| 38 | Corticosteroid therapy |  |
| 40 | Corticosteroid therapy |  |
| 43 | Corticosteroid therapy |  |
| 45 | Active malignancy |  |
| 47 | HIV infection |  |
| 52 | Corticosteroid therapy |  |
| 53 | Corticosteroid therapy |  |
| 58 | Solid organ transplantation |  |
| 62 | Corticosteroid therapy |  |
| 63 | Cancer chemotherapy |  |
| 65 | Corticosteroid therapy |  |
| 74 | Corticosteroid therapy |  |
| 75 | Immunosuppressive therapy |  |
| 82 | Immunosuppressive therapy |  |
| 86 | Corticosteroid therapy |  |
| 91 | Hematopoietic stem cell transplantation |  |
| 92 | Immunosuppressive therapy |  |
| 107 | Corticosteroid therapy |  |
| 109 | Corticosteroid therapy |  |
| 114 | Corticosteroid therapy |  |
| 116 | Corticosteroid therapy |  |
| 119 | Corticosteroid therapy |  |
| 120 | Solid organ transplantation |  |
| 147 | HIV infection |  |
| 148 | Corticosteroid therapy |  |
| 152 | Immunosuppressive therapy |  |
| 155 | Corticosteroid therapy |  |
| 156 | Corticosteroid therapy |  |
| 159 | Cancer chemotherapy |  |
| 167 | Active malignancy |  |
| 170 | Cancer chemotherapy |  |
| 171 | Immunosuppressive therapy |  |
| 178 | Immunosuppressive therapy |  |
| 179 | Cancer chemotherapy |  |
| 180 | Corticosteroid therapy |  |
| 182 | Corticosteroid therapy |  |
| 183 | Active malignancy |  |
| 188 | Corticosteroid therapy |  |
| 189 | Corticosteroid therapy |  |
| 197 | Corticosteroid therapy |  |
| 200 | Immunosuppressive therapy |  |
| 204 | Immunosuppressive therapy |  |
| 210 | Immunosuppressive therapy |  |
| 213 | Immunosuppressive therapy |  |
| 214 | Corticosteroid therapy |  |
| 218 | Active malignancy |  |
| 221 | Corticosteroid therapy |  |
| 224 | Immunosuppressive therapy |  |
| 226 | Corticosteroid therapy |  |
| 230 | Corticosteroid therapy |  |
| 232 | Corticosteroid therapy |  |
| 235 | Active malignancy |  |
| 238 | Immunosuppressive therapy |  |
| 239 | Immunosuppressive therapy |  |
| 245 | Corticosteroid therapy |  |
| 246 | Immunosuppressive therapy |  |
| 252 | Corticosteroid therapy |  |
| 255 | Corticosteroid therapy |  |
| 256 | Solid organ transplantation |  |
| 258 | Corticosteroid therapy |  |
| 261 | Corticosteroid therapy |  |
| 266 | Active malignancy |  |
| 267 | Solid organ transplantation |  |
| 281 | Immunosuppressive therapy |  |
| 286 | Corticosteroid therapy |  |
| 293 | Immunosuppressive therapy |  |
| 302 | Active malignancy |  |
| 304 | Immunosuppressive therapy |  |
| 305 | Active malignancy |  |
| 307 | Corticosteroid therapy |  |
| 311 | Immunosuppressive therapy |  |
| 312 | Corticosteroid therapy |  |
| 313 | Corticosteroid therapy |  |
| 315 | Corticosteroid therapy |  |
| 316 | Corticosteroid therapy |  |
| 318 | Active malignancy |  |
| 321 | Corticosteroid therapy |  |
| 322 | Corticosteroid therapy |  |
| 324 | Immunosuppressive therapy |  |
| 328 | Corticosteroid therapy |  |
| 329 | Corticosteroid therapy |  |
| 332 | Active malignancy |  |
| 333 | Corticosteroid therapy |  |
| 336 | Immunosuppressive therapy |  |
| 337 | Immunosuppressive therapy |  |
| 343 | Corticosteroid therapy |  |
| 346 | Corticosteroid therapy |  |
| 347 | Corticosteroid therapy |  |
| 354 | Corticosteroid therapy |  |
| 355 | Corticosteroid therapy |  |
| 357 | Corticosteroid therapy |  |
| 358 | Solid organ transplantation |  |
| 360 | Active malignancy |  |
| 362 | Corticosteroid therapy |  |
| 364 | Corticosteroid therapy |  |
| 365 | Active malignancy |  |
| 366 | Immunosuppressive therapy |  |
| 367 | Corticosteroid therapy |  |
| 369 | Corticosteroid therapy |  |
| 370 | Corticosteroid therapy |  |
| 372 | Corticosteroid therapy |  |
| 374 | Corticosteroid therapy |  |
| 383 | Immunosuppressive therapy |  |
| 387 | Corticosteroid therapy |  |
| 389 | Cancer chemotherapy |  |
| 390 | Active malignancy |  |
| 391 | Active malignancy |  |
| 392 | Active malignancy |  |

**Table S2** The laboratory testing results of patients with severe community-acquired pneumonia (immunocompromised vs immunocompetent patients)

| Variable | Total | Immunocompromised | Immunocompetent | *P* Value |
| --- | --- | --- | --- | --- |
|  | (n = 393) | (n = 119) | (n = 274) |  |
| White blood cell (×10^9^/L, median, IQR) | 9.8 (6.6 - 14.1) | 9.1 (5.9 - 11.9) | 10.2 (6.8 - 14.6) | 0.030 |
| Neutrophils (×10^9^/L, median, IQR) | 8.5 (5.7 - 12.4) | 7.9 (5.2 - 10.2) | 8.9 (5.9 - 13.2) | 0.005 |
| Lymphocytes (×10^9^/L, median, IQR) | 0.6 (0.4 - 1.0) | 0.5 (0.3 - 0.9) | 0.7 (0.4 - 1.1) | 0.044 |
| Hemoglobin (g/L, mean ± SD) | 112.1 ± 24.6 | 104.0 ± 21.5 | 115.6 ± 25.0 | < 0.001 |
| Platelet (×10^9^/L, median, IQR) | 169.0 (113.0 - 231.0) | 150.0 (93.5 - 217.5) | 175.0 (120.2 - 242.5) | 0.038 |
| Albumin (g/L, median, IQR) | 30.0 (27.0 - 34.0) | 29.0 (26.0 - 33.0) | 31.0 (28.0 - 34.0) | < 0.001 |
| Creatinine (μmol/L, median, IQR) | 71.7 (54.1 - 102.4) | 71.7 (52.2 - 101.5) | 71.8 (54.6 - 102.4) | 0.804 |
| PT (sec, median, IQR) | 15.1 (14.2 - 16.5) | 15.2 (14.4 - 16.5) | 15.1 (14.2 - 16.7) | 0.115 |
| APTT (sec, median, IQR) | 44.8 (39.2 - 52.4) | 44.5 (37.6 - 53.2) | 45.2 (39.8 - 52.3) | 0.888 |
| D dimer (mg/L, median, IQR) | 3.2 (1.7 - 8.1) | 2.8 (1.5 - 7.8) | 3.4 (1.9 - 8.4) | 0.236 |
| Fibrinogen (g/L, mean ± SD) | 5.7 ± 2.1 | 5.6 ± 2.2 | 5.7 ± 2.0 | 0.647 |
| PaO_2_/FiO_2_ (mmHg, median, IQR) | 147.1 (98.6 - 206.9) | 145.3 (96.5 - 205.8) | 148.6 (102.2 - 206.6) | 0.276 |
| FiO_2_ (median, IQR) | 0.6 (0.4 - 0.8) | 0.5 (0.4 - 0.8) | 0.6 (0.4 - 0.8) | 0.829 |
| Procalcitonin (ng/mL, median, IQR) | 1.0 (0.4 - 5.1) | 0.8 (0.4 - 3.4) | 1.2 (0.4 - 6.0) | 0.020 |
| Lactate (mmol/L, median, IQR) | 1.5 (1.1 - 2.0) | 1.5 (1.1 - 2.1) | 1.5 (1.0 - 1.9) | 0.238 |
| CD4 T cells (cells/mL, median, IQR) | 262.5 (153.0 - 419.8) | 201.5 (116.0 - 351.0) | 286.0 (177.2 - 458.2) | 0.001 |

**Abbreviations:** IQR, interquartile range; SD, standard deviation; APTT, activated partial thromboplastin clotting time; PT, prothrombin time; PaO2/FiO2, the ratio of arterial oxygen partial pressure to fractional inspired oxygen; FiO2, fractional inspired oxygen.

**Table S3** Distribution of pathogens in patients with severe community-acquired pneumonia (immunocompromised vs immunocompetent patients)

| **Pathogens** | **Immunocompromise (n = 99)** | **Immunocompetent (n = 189)** |
| --- | --- | --- |
| Influenza A virus | 9 | 82 |
| Aspergillus | 23 | 44 |
| Atypical pathogens | 4 | 29 |
| Influenza B virus | 6 | 15 |
| Klebsiella pneumoniae | 5 | 13 |
| Pseudomonas aeruginosa | 5 | 13 |
| Adenovirus | 0 | 10 |
| Streptococcus pneumoniae | 0 | 9 |
| Respiratory syncytial virus | 2 | 7 |
| Cytomegalovirus | 47 | 6 |
| Mycobacterium tuberculosis | 3 | 6 |
| Staphylococcus aureus | 9 | 4 |
| Escherichia coli | 2 | 3 |
| Pneumocystis jirovecii | 55 | 0 |
| Nontuberculous mycobacteria | 2 | 0 |
| Nocardia spp. | 2 | 0 |

| **Table S4** Univariable analysis of ICU mortality in immunocompromised patients with severe community acquired pneumonia (Variables entered into the multivariate logistic regression model with *P* values < 0.1) | | |  |
| --- | --- | --- | --- |
| Variable | Univariate logistic analysis | |  |
|  | OR (95% CI) | *P* value |  |
| Age |  |  |  |
| <45 | Reference |  |  |
| 45 ≤ age ＜ 65 | 1.917 (0.635 - 5.781) | 0.248 |  |
| ≥ 65 | 3.889 (1.268 - 11.928) | 0.018 |  |
| SOFA score | 1.303 (1.133 - 1.498) | < 0.001 |  |
| APACHE II score | 1.057 (0.997 - 1.120) | 0.062 |  |
| Neutrophils (×10^9^/L) | 1.082 (1.000 - 1.171) | 0.050 |  |
| Lymphocytes (× 10^9^/L) |  |  |  |
| Lymphocyte ≥ 0.8 | Reference |  |  |
| Lymphocytes <0.8 | 3.739 (1.631 - 8.570) | 0.002 |  |
| Platelet (× 10^9^/L) |  | ≥ |  |
| Platelet ≥ 100 | Reference |  |  |
| Platelet < 100 | 4.167 (1.734 - 10.013) | 0.001 |  |
| D dimer (mg/L) | 1.172 (1.075 - 1.278) | < 0.001 |  |
| Fibrinogen (g/L) | 0.785 (0.658 - 0.937) | 0.007 |  |
| CD4 (cells/mL) |  |  |  |
| CD4 ≥ 200 | Reference |  |  |
| CD4 < 200 | 4.094 (1.763 - 9.505) | 0.001 |  |
| IMV on ICU admission |  |  |  |
| No | Reference |  |  |
| Yes | 2.294 (0.928 - 5.671) | 0.072 |  |
| PaO_2_/FiO_2_ (mmHg) | 0.994 (0.990 - 0.998) | 0.004 |  |
| FiO_2_ |  |  |  |
| < 0.5 | Reference |  |  |
| 0.5 ≤ FiO_2_ < 0.7 | 2.600 (0.927 - 7.293) | 0.069 |  |
| ≥ 0.7 | 7.200 (2.825 - 18.348) | < 0.001 |  |
| Chronic kidney disease | 0.408 (0.172 - 0.971) | 0.043 |  |
| Lactate (mmol/L) | 2.006 (1.183 - 3.403) | 0.010 |  |
| **Abbreviations:** ICU, intensive care unit; SOFA, sequential organ failure assessment; APACHE, acute physiology and chronic health evaluation scoring system; FiO_2_, fractional inspired oxygen; PaO_2_/FiO_2_, the ratio of arterial oxygen partial pressure to fractional inspired oxygen; IMV, invasive mechanical ventilation; OR, Odds ratio; CI, confidence interval. | | |  |
|  |  |  |  |
|  |  |  |  |

| **Table S5** Univariable analysis of ICU mortality in immunocompetent patients with severe community acquired pneumonia (Variables entered into the multivariate logistic regression model with P values < 0.1) | | |  |
| --- | --- | --- | --- |
| Variable | Univariate logistic analysis | |  |
|  | OR (95% CI) | *P* value |  |
| Age |  |  |  |
| <45 | Reference |  |  |
| 45 ≤ age ＜ 65 | 1.448 (0.665 - 3.155) | 0.351 |  |
| ≥ 65 | 2.721 (1.301 - 5.690) | 0.008 |  |
| Season |  |  |  |
| Spring and Autumn | Reference |  |  |
| Summer | 1.032 (0.513 - 2.076) | 0.930 |  |
| Winter | 1.723 (0.992 - 2.995) | 0.053 |  |
| Days from illness onset to ICU | 1.097 (1.049 - 1.148) | < 0.001 |  |
| SOFA score | 1.150 (1.076 - 1.230) | < 0.001 |  |
| APACHE II score | 1.097 (1.056 - 1.140) | < 0.001 |  |
| Neutrophils (×10^9^/L) | 1.049 (1.011 - 1.089) | 0.012 |  |
| Lymphocytes (× 10^9^/L) |  |  |  |
| Lymphocyte ≥ 0.8 | Reference |  |  |
| Lymphocytes <0.8 | 1.689 (1.020 - 2.799) | 0.042 |  |
| Platelet (× 10^9^/L) |  |  |  |
| Platelet ≥ 100 | Reference |  |  |
| Platelet < 100 | 2.538 (1.347 - 4.781) | 0.004 |  |
| D dimer (mg/L) | 1.060 (1.018 - 1.103) | 0.004 |  |
| Fibrinogen (g/L) | 0.858 (0.754 - 0.977) | 0.020 |  |
| CD4 (cells/mL) |  |  |  |
| CD4 ≥ 200 | Reference |  |  |
| CD4 < 200 | 1.754 (0.973 - 3.161) | 0.062 |  |
| IMV on ICU admission |  |  |  |
| No | Reference |  |  |
| Yes | 1.839 (1.118 - 3.023) | 0.016 |  |
| PaO_2_/FiO_2_ (mmHg) | 0.994 (0.990 - 0.998) | 0.004 |  |
| FiO_2_ |  |  |  |
| < 0.5 | Reference |  |  |
| 0.5 ≤ FiO_2_ < 0.7 | 1.806 (0.899 - 3.625) | 0.097 |  |
| ≥ 0.7 | 4.189 (2.199 - 7.981) | < 0.001 |  |
| Lactate (mmol/L) | 1.290 (1.050 - 1.585) | 0.015 |  |
| **Abbreviations:** ICU, intensive care unit; SOFA, sequential organ failure assessment; APACHE, acute physiology and chronic health evaluation scoring system; FiO_2_, fractional inspired oxygen; PaO_2_/FiO_2_, the ratio of arterial oxygen partial pressure to fractional inspired oxygen; IMV, invasive mechanical ventilation; OR, Odds ratio; CI, confidence interval. | | |  |
|  |  |  |  |
|  |  |  |  |

| **Table S6** Risk factors for ICU mortality in immunocompetent patients with severe community acquired pneumonia | | |  |
| --- | --- | --- | --- |
|  |  |  |  |
| Variable | Multivariate logistic analysis | |  |
|  | OR (95% CI) | *P* value |  |
| Days from illness onset to ICU | 1.101 (1.039 - 1.166) | 0.001 |  |
| Platelet (×10^9^/L) |  |  |  |
| Platelet ≥ 100 |  |  |  |
| Platelet < 100 | 2.729 (1.191 - 6.256) | 0.018 |  |
| FiO_2_ |  |  |  |
| < 0.5 | Reference |  |  |
| 0.5 ≤ FiO_2_ < 0.7 | 1.601 (0.655 - 3.916) | 0.302 |  |
| ≥ 0.7 | 3.372 (1.433 - 7.932) | 0.005 |  |
| Abbreviations: ICU, intensive care unit; APACHE, acute physiology and chronic health evaluation scoring system; FiO_2_, fractional inspired oxygen; OR, Odds ratio; CI, confidence interval. | | |  |

1. Peng JM, Du B, Qin HY, Wang Q, Shi Y. Metagenomic next-generation sequencing for the diagnosis of suspected pneumonia in immunocompromised patients. J Infect. (2021) 82: 22-27. doi:10.1016/j.jinf.2021.01.029

2. Zhan Y, Xu T, He F, Guan WJ, Li Z, Li S, et al. Clinical Evaluation of a Metagenomics-Based Assay for Pneumonia Management. Front Microbiol. (2021) 12: 751073. doi:10.3389/fmicb.2021.751073

3. Li H, Gao H, Meng H, Wang Q, Li S, Chen H, et al. Detection of Pulmonary Infectious Pathogens From Lung Biopsy Tissues by Metagenomic Next-Generation Sequencing. Front Cell Infect Microbiol. (2018) 8: 205. doi:10.3389/fcimb.2018.00205

4. Qian YY, Wang HY, Zhou Y, Zhang HC, Zhu YM, Zhou X, et al. Improving Pulmonary Infection Diagnosis with Metagenomic Next Generation Sequencing. Front Cell Infect Microbiol. (2020) 10: 567615. doi:10.3389/fcimb.2020.567615

5. Zhang F, Chen J, Huang H, Deng X, Zhang W, Zeng M, et al. Application of metagenomic next-generation sequencing in the diagnosis and treatment guidance of Pneumocystis jirovecii pneumonia in renal transplant recipients. Eur J Clin Microbiol Infect Dis. (2021) 40: 1933-1942. doi:10.1007/s10096-021-04254-x

6. Qu J, Zhang J, Chen Y, Huang Y, Xie Y, Zhou M, et al. Aetiology of severe community acquired pneumonia in adults identified by combined detection methods: a multi-centre prospective study in China. Emerg Microbes Infect. (2022) 11: 556-566. doi:10.1080/22221751.2022.2035194
